# Immunogenicity and Efficacy of Single Antigen Gp63, Polytope and PolytopeHSP70 DNA Vaccines against Visceral Leishmaniasis in Experimental Mouse Model

**DOI:** 10.1371/journal.pone.0007880

**Published:** 2009-12-02

**Authors:** Rakhee Sachdeva, Akhil C. Banerjea, Nancy Malla, Mohan Lal Dubey

**Affiliations:** 1 Department of Parasitology, Postgraduate Institute of Medical Education and Research, Chandigarh, India; 2 National Institute of Immunology, New Delhi, India; Sabin Vaccine Institute, United States of America

## Abstract

Polytope approach of genetic immunization is a promising strategy for the prevention of infectious disease as it is capable of generating effective cell mediated immunity by delivering the T cell epitopes assembled in series. Leishmaniasis is a significant world wide health problem for which no vaccine exists. In this study we have compared immunogenicity and efficacy of three types of DNA vaccines: single antigen Gp63 (Gp63/pcDNA), polytope (Poly/pcDNA) and Polytope fused with hsp70 (Poly/hsp/pcDNA) against visceral leishmaniasis in susceptible BALB/c mice. Mice vaccinated with these plasmids generated strong Th1 immune response as seen by dominating IFN-γ over IL-10 cytokine. Interestingly, cytotoxic responses generated by polytope DNA plasmid fused with hsp70 of *Leishmania donovani* were significantly higher when compared to polytope and single antigen Gp63 vaccine. Challenge studies revealed that the parasite load in liver and spleen was significantly lower with Poly/hsp/pcDNA vaccination compared to other vaccines. Therefore, our study indicates that polytope DNA vaccine is a feasible, practical and effective approach for visceral leishmaniasis.

## Introduction

Poly-epitope based DNA immunization approach has an excellent ability to induce T cell responses. Polytope DNA vaccine encodes multiple continuous T cell epitopes which induce specific Cytotoxic T lymphocyte (CTL) responses or T helper (Th) responses to individual epitope. Earlier studies have shown that each epitope in polytope constructs is processed, presented and is immunogenic in animals with an appropriate MHC background[1]. The polytope approach allows epitopes restricted by a range of different MHC alleles to be combined, making feasible the construction of epitope based vaccines that cover wider HLA diversity of the target population[1]. Experimental Polytope vaccines have been developed for number of pathogens such as HIV/AIDS, HBV and cancers[2]–[6]. The main advantage of this strategy is its ability to eliminate portions of the antigen that may cause harmful immune responses[7]. Malarial parasites exhibit certain regions that may decoy responses thereby eliciting antibodies of irrelevant specificities and diverting protective responses. Gilbert et al[8] generated a polytope vaccine against *Plasmodium Sps.* that contained a string of 15 defined cytotoxic T lymphocyte (CTL) epitopes from *Plasmodium* species which primed protective CTL responses in mice following a single administration without adjuvant. The immunogenicity of HIV polytope vaccine containing multiple HLA A2 HIV CD8+ cytotoxic T-cell epitopes was shown by Woodberry et al[9].

Visceral Leishmaniasis (VL) is a major public health problem with significant morbidity and mortality worldwide[10]. Cellular immune mechanisms are critical for recovery from VL and for protection from re-infection in both human and mice[10]. Although leishmanial infections induce strong humoral responses, the role of the elevated antileishmanial antibodies in kala-azar patients towards protection and pathogenesis is still unclear[11]. An experimental study postulated that IgG not only fails to provide protection against this intracellular pathogen, but it actually contributes to disease progression[12], [13]. Passive administration of antileishmanial IgG antibodies resulted in larger lesions in Balb/c mice with greater amount of IL-10 production[12]. Thus, it appears that anti-leishmanial antibodies or humoral immunity does not play a protective role in the control of the disease.

Early classical experiments established that CD4+ T cells are crucial for resistance, whereas CD8+ T cells seem to participate more in generation of immune memory and as effector cells for parasite elimination[14]. However, recent studies have suggested that CD8+ T cells may be involved in the clearance of primary infection[15]. In rodent models, the Th1/Th2 paradigm is important in determining the outcome of murine *L. major* infection[16]. This dichotomy is not well demarcated during murine *L. donovani/L. chagasi* infection in which curative type1 responses may be suppressed by IL-10 and TGF-β [17]. Protective immunity against VL as in case of CL (Cutaneous leishmaniasis) is dependent on IL-12 driven type1 response characterized by IL-2 and IFN-γ production, which results in the induction of parasite killing[18]. Regarding immune responses in human acute visceral leishmaniasis, the cytokine profile is high production of IL-4 and IL-10 and low IL-2 and IFN- γ production[19].

Recent advances in cellular immunity have greatly increased the potential of peptides as immunogens for CTLs. These include the demonstration that CTLs can recognize small antigenic peptides of 8–10 amino acids in length, which complexed with MHC class I molecules and are expressed on the surface of infected or cancer cells to be presented to T-cell receptors[20], [21]. Targeting of dominant epitopes may be an effective way to overcome CTL tolerance [22] and to allow the immune response to focus on highly conserved epitopes[23].

The best candidates for designing a vaccine are the proteins required for parasite survival or adhesion of parasites to host cells, have low mutation rates and have conserved epitopes. Gp63, a glycoprotein of 63 kilo Dalton that occurs on the surface of *Leishmania* was the first candidate from *Leishmania major* used for DNA vaccine[24]. It has been demonstrated earlier that *L. donovani* contains more chromosomal mini-exon gene sequences than *L. major* which contribute to its increased virulence[25]. The structure of gp63 gene of *L. donovani* contains 7 tandem repeats and each repeat contains 1.8 kb coding region and 1.3 kb intergenic region[26]. Russo et al[27] has identified human T cell epitopes in both *L.major* and *L. chagasi* gp63. Some of these T cell epitopes induced proliferative and IFN-γ responses in cells from infected individuals. Two of the peptide epitopes from *L. major* and *L. chagasi* gp63 were capable of generating *Leishmania* specific T cell lines *invitro* [27]. We have identified corresponding 2 human T cell epitopes and 7 murine T cell epitopes in the coding region of *Leishmania donovani*, Gp63 gene (M60048) using NCBI BLAST (Basic Local Alignment Search Tool). In the present study, a polytope DNA vaccine was prepared using these two immunogenic human T cell epitopes of *Leishmania donovani*, Gp63 gene which were analogous to the epitopes identified by Russo et al, [27] in *L.major* and *L. chagasi* gp63 gene. Out of these two immunogenic human T cell epitopes, one is both human as well as murine T cell epitope [27].

Srivastava et al[28] have demonstrated that heat shock protein (hsp) strongly enhances the immune reaction to tumor-associated antigens. Several studies showed that hsp70 isolated from tumor cells was able to induce specific CTL responses capable of protecting against tumor growth and viral infection [29]. *Mycobacterium tuberculosis* hsp70 has been found to be a powerful antigen containing multiple B- and T-cell epitopes and to induce CTL response in dendritic cells in a CD4 Th-independent manner[3]. In fact, Suzue et al [30] produced and purified a recombinant HIV-I p24-hsp70 fusion protein and demonstrated that it could elicit both humoral and cellular responses against HIV-1 p24 in the absence of adjuvant[31]. The MHC class I in conjunction with hsp70 has been shown to generate CTL responses[32]. Wang et al[33] suggested that CD40 was a cellular receptor for TBhsp70. The CD40/CD40L interaction also leads to production of inflammatory cytokines, such as TNF-α, IL-1, IL-6, IL-12 and RANTES [34]. Indeed, recent studies demonstrated a central role for ligation of CD40 on macrophages and dendritic cells in the induction of MHC class I-restricted antigen specific CD8+ T-cell responses and protective immunity[35].

In the present study, three vaccine formulations i.e. single antigen Gp63 DNA vaccine (coding region of Gp63 gene of *L.donovani*), Polytope DNA vaccine and Polytope DNA vaccine fused to hsp70 molecule of *Leishmania donovani* were compared for their immunogenicity and efficacy in a mouse model.

## Materials and Methods

### Animals

3–4 weeks old, female BALB/c mice weighing 15–18 gm were obtained from the Central Animal Facility, National Institute of Pharmaceutical Education and Research, Mohali, India. All animals were housed and used in the departmental animal house in accordance with institutional guidelines.

The study was reviewed and approved by the Institute Ethical Committee of Postgraduate Institute of Medical Education and Research, Chandigarh, India.

### Culture of Leishmania promastigotes

AG83 strain of *Leishmania donovani* was used in the present study. The parasite was passaged in BALB/c mice before the experiments to maintain the virulence. The amastigotes obtained from spleens of BALB/c were suspended in DMEM with 30% Fetal Calf Serum (FCS). This suspension was incubated at 22°C for 48 to 72 h. Freshly transformed promastigotes were checked under the microscope for their morphology and number. The suspension was centrifuged at 1000 rpm for 10 min at 4°C to remove splenic debris and promastigotes were pelleted down at 5000 rpm for 15 min at 4°C. These promastigotes were maintained in DMEM with 10% FCS at 22°C. Subculturing was done on every fourth day when the promastigotes attained stationary phase of growth.

### Cloning and expression of Gp63 gene

PCR cloning was carried out after PCR amplification of coding region of Gp63 gene of *Leishmania donovani* using following pair of primers.

forward 5′ GCA GCC GGA TCC ATG TCC GTC GAC AGC AGC AGC 3′

reverse 5′ GCG GCC AAG CTT CAC GCC ATC ACC ACC CGT CCT 3′

The 1.8 kb PCR product obtained was digested with *BamHI* and *HindIII* and ligated into pET-30a vector (Novagen, Madison, USA) for Recombinant Protein and into pcDNA3.1 vector (Invitrogen, USA) for DNA vaccine. The ligated product was transformed into *E. coli* BL21 and DH5α cells respectively for pET30a vector and pcDNA3.1 vector. Transformants were analyzed by restriction digestion and the positive clone was sequenced to confirm the presence of Gp63 insert in proper reading frame (Data S1). The positive clones were designated pET30/Gp63 and pcDNA/Gp63. Transformed BL-21 cells were amplified by growing on LB broth medium supplemented with 50 µg/ml kanamycin. The transformed cells were grown to log phase (OD_600_ = 0.6 to 1.0) and IPTG was added to a final concentration of 0.4 mM. The culture was grown at 37°C in a shaker incubator and 1 ml aliquots were collected at each hour. The same number of cells from each aliquot was loaded on an SDS-PAGE gel and checked for recombinant protein expression (pET-system manual, 1992, Novagen, Madison, USA).

The *invitro* expression of the inserted Gp63 gene in eukaryotic expression vector pcDNA3.1 was checked by transfection of gp63/pcDNA construct in mammalian cells i.e. 293 cells & J774A.1 macrophage cells. The expression was confirmed by Qualitative reverse transcription PCR (RT-PCR). Total RNA was isolated from the transfected cells using TRIZOL reagent (Invitrogen, USA) according to manufacturer's instructions. RT-PCR was performed using RT-PCR kit (Promega, Madison, USA) using 3 µg of each of the RNA sample.

### Purification of recombinant GP63 (rGP63) protein by urea gradient

The induced *E. coli* (BL21) culture cells were collected and resuspended in 10 ml of TNE (Tris-Cl, NaCl, EDTA) buffer followed by addition of 10 mg of lysozyme. It was then kept on ice for 30 min. The lysed cells were centrifuged at 4000 g for 10 min sat 4°C and pellet and supernatant fractions were separated. The induced protein was found to be in the pellet fraction. The pellet was treated for purification of recombinant Gp63 protein by urea gradient method using 2 M, 4 M and 8 M urea concentrations as described by Sukumaran et al[36]. The positive fractions were identified by SDS-PAGE and dialysed extensively against slowly decreasing concentrations of urea in PBS. Protein was further purified and concentrated by using Centricon-30 filters (Amicon, USA).

### Preparation of DNA vaccines

#### *Single antigen DNA vaccine*

The coding region of gp63 gene of *Leishmania donovani* (1.8 kb) cloned in pcDNA3.1 plasmid was used as single antigen DNA vaccine.

#### *Polytope DNA vaccines*

**Figure 1:**
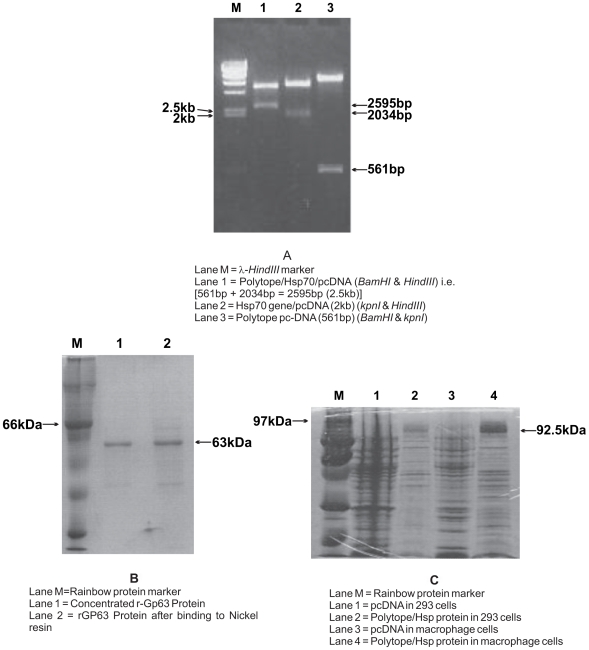
Construction of vaccine plasmids for DNA vaccination. (**A**) Polytope Vaccine design: The DNA sequence coding for the polytope antigen contained Kozak sequence, IgG κ leader sequence, PADRE sequence and two T cell epitopes. Each epitope was separated with AAA nucleotides encoding lysine. This polytope gene was made by joining seven overlapping oligonucleotides using splicing by overlap extension and PCR and was cloned into BamHI and kpnI restriction sites of pcDNA3.1 vector. The Poly/pcDNA encoded Polytope antigen (Polytope DNA vaccine), Poly/hsp/pcDNA encoded hsp70-fused polytope antigen (Poly/hsp DNA vaccine). (**B**) Nucleotide sequence of final product of Polytope DNA which showed 100% homology to designed sequence. (**C**) Restriction digestion of vaccine plasmid constructs to confirm the presence of insert gene sequences. (**D**) SDS-PAGE (12%) analysis of *invitro* expression of Polytope antigen fused with hsp70 in J774A.1 macrophages and 293 cells.

pcDNA3.1(−) vector was used to construct polytope DNA vaccine plasmids. The T cell epitopes included in the vaccine were: two immunogenic gp63 T cell epitopes PT1 and PT7 which we have identified in AG83 strain of *L. donovani* and one universal Th Pan DR epitope [(PADRE) sequence] [3]. PADRE is a synthetic Th epitope engineered by introducing anchor residues for the different DR motifs of MHC II into a polyalanine backbone[37] and the resulting peptide binds a variety of DR molecules as well as certain mouse class II alleles, including I-Ab, I-Ed and I-Ek. Each epitope was separated by AAA nucleotides encoding lysine. This artificial polytope antigen also contained IgG κ chain leader sequence (used as a signal peptide) and a Kozak sequence which was inserted at the 5′-terminal of signal peptide as the ribosome-binding site[3]. The whole designed sequence was divided into seven equal fragments, averaging 75 nucleotides in length (Fig. 1A). The primers were designed with 15 oligonucleotide overlaps between consecutive fragments. These seven fragments were spliced together using the primers by overlap extension and PCR techniques (Li et al 2005) to obtain a final polytope antigen gene (Fig. 1A). All the oligomers and primers were synthesized commercially with 2–5 OD (40 nm) and were machine grade purified.

For enhancing CTL activity, hsp70 gene of *Leishmania donovani* was fused to the C-terminal of polytope antigen gene and was inserted into the pcDNA vector[34]. Hsp70 gene was amplified from genomic DNA of *Leishmania donovani* using following pair of primers:

Forward primer

5′ CGG CTT CTG CTG GCT TTG GTG CTG GCT TTG GTT TGG TAC CAT

GAC ATT CGA CGG CGC C 3′

Reverse primer

5′ CCC AAG CTT GGG TTA GTC GAC CTC CTC GAC CTT 3′

*BamHI*, *KpnI* and *HindIII* restriction sites were included in the primers for cloning purposes. The positive clones were confirmed by restriction digestion with the respective enzymes (Fig. 1C). Both the constructs i.e. Polytope/pcDNA and Polytope/Hsp/pcDNA were sequenced to confirm the correct orientation of the introduced sequences. The plasmid preparation for immunization experiments was undertaken by using the EndoFree Plasmid Giga kit (Qiagen, Germany). The DNA purified with EndoFree plasmid kits contains only negligible amounts of endotoxin (<0.1 EU/µg plasmid DNA).

### Immunization Schedule

Immunization schedule followed was as reported earlier by Yang et al [38] with slight modifications. A total of 18 mice were used for each vaccination and control group1 while 12 mice were employed as normal controls (control group 2) for both immunological and parasitological assays.

As illustrated in table 1, there were 3 parenteral DNA vaccines used in the study i.e. single antigen Gp63 DNA vaccine (Gp63/pcDNA - groups 1, 2), Polytope DNA vaccine (Polytope/pcDNA-groups 3, 4) and Polytope DNA vaccine fused with hsp molecule (Polytope/hsp/pcDNA-groups 5, 6) which were administered intramuscularly [24] in three doses of 100 µg each at weekly intervals followed by a final booster on day 21 with the respective DNA vaccine constructs to groups 1, 3 & 5 and with recombinant Gp63 protein by intraperitoneal route [39] to groups 2, 4 & 6. Two control groups were included: Control group 1 included vector plasmid pcDNA alone while Control group 2 comprised of un-inoculated healthy mice as normal controls.

**Table 1:** Vaccination schedule for immunogenicity and efficacy studies in BALB/c mice.

Time of Immunization And Challenge	Group 1 Gp63 DNA	Group 2 Gp63/PB	Group 3 Poly DNA	Group 4 Poly/PB	Group 5 Poly/Hsp	Group 6 Poly/Hsp/PB	Control group 1 pcDNA	Normal Control group 2 PBS
0 day (1^st^ dose)	Gp63/pcDNA (100 µg) (i.m.)	Gp63/pcDNA (100 µg) (i.m.)	Polytope/pcDNA (100 µg) (i.m.)	Polytope/pcDNA (100 µg) (i.m.)	Polytope/hsp/pcDNA (100 µg) (i.m.)	Polytope/hsp/pcDNA (100 µg) (i.m.)	Vector plasmid 100 µg (i.m.)	PBS
7^th^ day (2^nd^ dose)	Gp63/pcDNA (100 µg) (i.m.)	Gp63/pcDNA (100 µg) (i.m.)	Polytope/pcDNA (100 µg) (i.m.)	Polytope/pcDNA (100 µg) (i.m.)	Polytope/hsp/pcDNA (100 µg) (i.m.)	Polytope/hsp/pcDNA (100 µg) (i.m.)	Vector plasmid 100 µg (i.m.)	PBS
14^th^ day (3^rd^ dose)	Gp63/pcDNA (100 µg) (i.m.)	Gp63/pcDNA (100 µg) (i.m.)	Polytope/pcDNA (100 µg) (i.m.)	Polytope/pcDNA (100 µg) (i.m.)	Polytope/hsp/pcDNA (100 µg) (i.m.)	Polytope/hsp/pcDNA (100 µg) (i.m.)	Vector plasmid 100 µg (i.m.)	PBS
21^st^ day (Booster)	Gp63/pcDNA (100 µg) (i.m.)	Recombinant Gp63 protein (i.p)	Polytope/pcDNA (100 µg) (i.m.)	Recombinant Gp63 protein (i.p.)	Polytope/hsp/pcDNA (100 µg) (i.m.)	Recombinant Gp63 protein (i.p.)	Vector plasmid 100 µg (i.m.)	PBS
Challenge 35^th^ day (2weeks after booster)	2×10^7^ promastigote (i.v.)	2×10^7^ promastigote (i.v.)	2×10^7^ promastigote (i.v.)	2×10^7^ promastigote (i.v.)	2×10^7^ promastigote (i.v.)	2×10^7^ promastigote (i.v.)	2×10^7^ promastigote (i.v.)	2×10^7^ promastigote (i.v.)

### Immunogenicity assays

The Immunogenicity of vaccines was studied by splenocyte proliferation, cytokine production and cytotoxicity assays. All the assays were performed on post immunization days 21, 35 and 63 (4 weeks after challenge) in all the groups of animals as described in Table 1. To see the effect of challenge infection on immunological parameters, unchallenged mice in each vaccine group were also included on day 63.

### Splenocyte proliferation assay

Spleens were removed from mice under aseptic conditions on a sterile dish containing DMEM medium. Single cell suspensions were prepared by grinding the spleen using an autoclaved mesh. 5–10 ml of DMEM medium was added to it and the contents were mixed to homogeneity. The dish was kept undisturbed for two minutes and the clear supernatant was pipetted out slowly. Cells were pelleted by centrifugation at 4°C at 250 g (Sorvall RC-5 centrifuge, HB-4 rotor) for 10 min. The pellet containing erythrocytes and splenocytes were collected. The pellet was washed once with 0.9% ammonium chloride to lyse the erythrocytes. The remaining cells i.e. splenocytes from each mouse in a group were pooled were resuspended to a density of 2.5×10^6^ cells/ml in DMEM containing 10% FCS and 0.05 µM 2-mercaptoethanol, then divided into 200 µl aliquots (5×10^5^ cells) in 1.5 ml eppendorf tubes. The splenocytes were re-stimulated with 1, 5, 10 µg recombinant GP63 antigen (rGP63 protein vaccine). These cells were incubated for 3-days at 37°C in atmosphere containing 5% CO_2_ and 95% humidity. Proliferation was measured by incorporation of 1 µCi of [^3^H]-thymidine over final 16 h of the 3 days of culture. The cells were harvested and taken in scintillation vials, scintillation fluid was added and counts were taken. Stimulation indices (S.Is) were calculated as the ratios of [^3^H]-thymidine incorporation in the presence of antigen versus the non-stimulated (medium alone) control. All assays were performed in triplicate, with four mice representing each group.

### Cytokine determination

IFN-γ, IL-2, IL-4 and IL-10 concentrations in cell culture supernatants were determined using CBA flex kit (BD Biosciences, Singapore) according to manufacturer instructions. The results were acquired using FACS CALIBUR (BD Biosciences, Singapore) and analyzed using FCAP software.

### Invitro cytotoxicity assays

Parasitized J774A.1 macrophage cells were used as target cells for the cytotoxicity assays. Macrophages were grown and plated at 2×10^5^/ml in six-well plates in Dulbecco MEM containing 10% FCS. Adherent cells were harvested on ice, and infected with late stationary phase *L. donovani* promastigotes. For infection, 10^5^ macrophages/well in a 24-well plate were centrifuged at 1,200 *g* for 1 h at RT to give a multiplicity of infection of 2 to 4 parasites per cell. At the end of infection, non-internalized parasites were separated from macrophages by washing with PBS. An aliquot of infected macrophages was stained with Giemsa to check the level of infection. Infected cells (10^6^ cells/ml) were also incubated with propidium iodide (100 µg/ml), a fluorescent dye which is excluded by viable cells and stains non-viable cells as red. Another dye fluorescein diacetate (FDA) which stain the viable cells showed green fluorescence when cells were incubated with 100 ng/ml of dye for 10 min at room temperature [40]. Microscopic examination verified that 70% of macrophages contained viable parasites. Cytolytic activity of splenocytes was evaluated by measuring lactate dehydrogenase (LDH) activity released into the medium, using the CytoTox96 nonradioactive assay (Promega, Madison, WI, USA) according to manufacturer instructions. Briefly, the parasitized macrophages (target cells) were co-cultured with splenocytes (effector cells) previously isolated at different time intervals from various vaccinated and unvaccinated groups of mice and stimulated with rGp63 protein and concanavalinA (Control). The effector (E) and target (T) cells were incubated for 4 h at E/T ratio of 1∶10. Cell lysis was determined by LDH release and it was quantified by measuring the absorbance at 490 nm. Maximum release was calculated from supernatants of cells that were lysed by addition of 10 µl of lysis solution (10x). The effector and target cells were included as separate controls for spontaneous release. The percentage of specific lysis was calculated as follows: [(Experimental – Spontaneous release)/(Total maximum release - Spontaneous release)] ×100.

### Protection studies

The efficacy of the vaccine preparations was determined by challenge infection of vaccinated and unvaccinated control mice with 2×10^7^ *Leishmania donovani* promastigotes intravenously, 2 weeks after the booster dose (day35, Table-1). The parasite loads in liver and spleen were determined after 4 weeks of challenge infection.

### Determining organ parasite load

After 4 weeks of challenge infection, mice were euthanized. The liver and spleen were aseptically removed and their impression smears were microscopically examined after fixing and staining the slides with Giemsa. In order to quantitate levels of infection, Leishman Donovan units (LDU) were calculated as: Number of amastigotes/Number of cell nuclei X weight of organ in milligrams [39]. Protection studies were performed using 6 mice per group. Results were evaluated by comparing the parasite load of test and control groups of mice.

### Statistical analysis

Data are expressed as the mean values ± S.D. of triplicate samples. The statistical significance of the differences between various groups was determined by PostHoc test and ANOVA. Differences were considered statistically significant for *p*<0.05.

## Results

### Epitope selection and vaccine plasmid construction

The single antigen Gp63 DNA (Gp63/pcDNA) and polytope (Poly/pcDNA, Poly/Hsp/pcDNA) vaccines were constructed as described above and used for DNA vaccination (Fig. 1A).

The Polytope vaccine preparations on sequencing showed 100% sequence identity with designed vaccine (Fig. 1B) which confirmed the correct orientation of the introduced sequences. The positive clones were confirmed by restriction digestion with respective enzymes (Fig. 1C). The prominent products of expected molecular masses 63kDa and 92kDa were expressed by eukaryotic cells (J774A.1 macrophages and 293) transfected with the plasmids Gp63/pcDNA and Polytope/Hsp/pcDNA (Fig. 1D) respectively but not by pcDNA3.1 vector transfected cells.

### Immunogenicity assays

For comparison of immunological and efficacy parameters between various vaccine groups and unvaccinated (control) groups, for control group the values of only PBS groups were included in the analysis as the values of pcDNA vector controls were not significantly different from that of PBS controls.

### Splenocyte Proliferation assay

**Figure 2:**
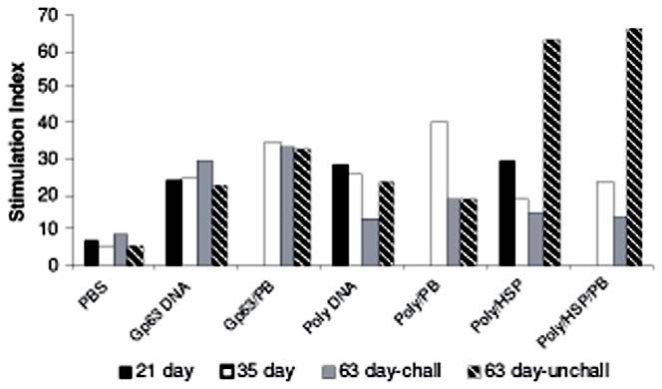
Splenocyte proliferation assay was performed on day 21, 35 and 63 following immunization and challenge infection in BALB/c mice. The mice were immunized with Gp63 single antigen DNA vaccine (Gp63/pcDNA) referred to as Gp63 DNA, Polytope DNA vaccine (Poly/pcDNA) referred as Poly DNA and Polytope/Hsp DNA vaccine (Poly/Hsp/pcDNA) referred as Poly/Hsp, thrice at weekly interval with and without rGp63 protein boosting as described in *Materials and Methods*. Splenocytes were stimulated with rGp63 protein or ConA (data not shown) and thymidine incorporation was determined. Stimulation Index represents the average counts per minute in stimulated cells divided by the average counts per minute in corresponding non-stimulated controls. The data is the mean of three experiments.

Splenocytes from all the vaccinated mice showed significantly higher proliferation than that of control group on all study days (day 21, 35 and 63) and highest response was seen with Poly/Hsp vaccine on day63 in unchallenged mice (p = 0.0001) (Fig. 2). Amongst challenged animals, the proliferative index on day 63 was highest in single antigen Gp63 DNA vaccine groups (Fig. 2). Protein boost significantly enhanced the stimulation index for Polytope DNA vaccine group (Poly/PB) on day35 (p = 0.001) but not in other two vaccine formulations (p>0.05) (Fig. 2).

### Cytotoxicity assay

**Figure 3:**
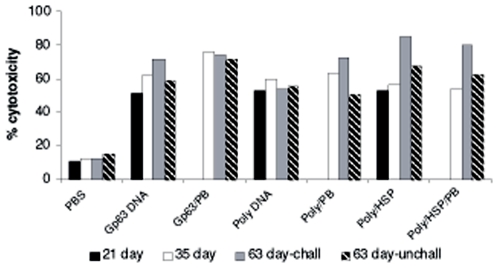
Percentage cytotoxicity of splenocytes isolated from various groups of immunized and control BALB/C mice. The splenocytes (Effector cells) were co-cultured with parasitized macrophages (Target cells) and cytotoxicity was measured as LDH release by spectrophotometric analysis. Statistically significant difference was seen in all the vaccinated groups when compared with controls (*p*<0.01). The results presented here are the mean values obtained from LDH release assay performed in triplicates.

In order to detect the cytolytic activity of splenocytes isolated from all the vaccinated groups of mice on day21, 35 and day63 (challenged & unchallenged), we tested their capacity to lyse the *Leishmania donovani* infected macrophage cells. Substantial levels of cytotoxicity were detected only in vaccinated animals (Fig. 3). There was no significant difference in the cytotoxicity of splenocytes on days 21 and 35 for single antigen Gp63 DNA and Polytope vaccine groups (Fig. 3). The Poly/hsp vaccine group showed highest cytotoxicity (85%) on day 63 (after 4 weeks of challenge infection) which was followed by Gp63 DNA vaccine (72%). In unchallenged mice on day63, polytope/hsp vaccine group showed significantly higher cytolytic activity compared to polytope group (p<0.01) but there was no significant difference in cytotoxicity between polytope/hsp and Gp63 DNA vaccine groups (Fig. 3). Protein boosting for Poly/DNA vaccine group significantly enhanced cytotoxicity (p<0.001) as seen on day 63 (Fig. 3). On the contrary, there was no significant effect of protein boost on single antigen Gp63 DNA and Poly/Hsp vaccinated groups.

### Cytokine responses

**Figure 4:**
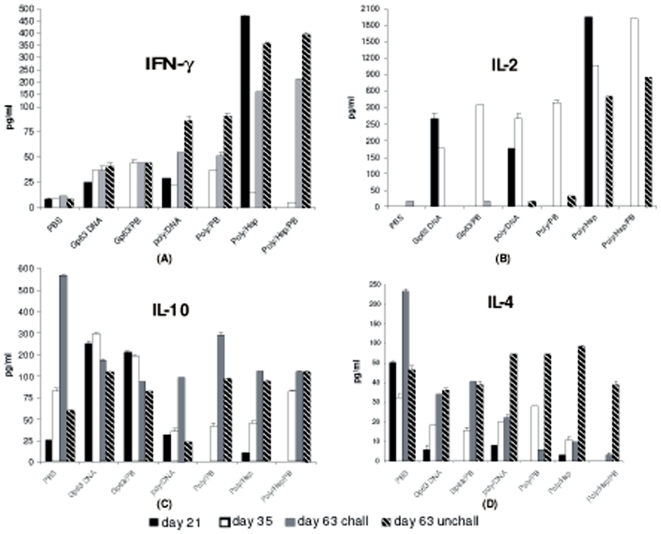
IFN-γ, IL-2, IL-4 and IL-10 production on day 21, day 35 and day 63 in all the vaccinated and control groups (6 mice per group) as shown in Fig. 2. Culture supernatants were collected and the cytokines were assayed by flow cytometry. Results presented represent the mean ± SD (vertical lines on bars) of the triplicate assays.

The cytokine responses in splenocytes were analyzed for each of the vaccine formulations (Fig. 4). Our results revealed a massive up-regulation of IFN-γ in cells isolated from vaccinated mice. Gp63 DNA vaccine elicited significant IFN-γ response on day 21which further increased on day 35. However, in this vaccine group, there was no significant increase in IFN-γ levels on day 63 in both challenged and unchallenged animals as compared to day 35 levels. Protein boosting significantly enhanced IFN-γ levels on day 35 & day 63 in challenged group for Gp63 DNA vaccine when compared with non-protein boost group. On day21, Poly/Hsp vaccine showed maximal IFN-γ levels (p = 0.0001) which declined massively on day35. Protein boost significantly enhanced IFN-γ on day 35 for Poly DNA vaccine group (p = 0.01). On day 63 in both challenged and unchallenged group, Poly/Hsp and Poly/Hsp/PB showed significantly higher levels (p = 0.001 to 0.0001) of IFN-γ compared to Poly DNA and Poly/PB groups (Fig. 4A).

Significant levels of IL-2 were seen increased on day 21 in Gp63 DNA vaccine group and on day 35 in Gp63/PB group. In contrast, IL-2 levels were almost undetectable after challenge infection. Polytope DNA vaccines with hsp70 showed significant IL-2 levels on all the days except on day 63 in challenged animals whereas Poly DNA vaccine without hsp70 showed significantly low IL-2 on day 63 both in challenged and unchallenged group. Protein boost significantly enhanced these levels on day 35 for both Gp63 DNA and both Polytope DNA vaccines (Fig. 4B).

Basal levels of IL-10 in PBS control group were significantly high after 28 days of challenge infection (on day 63) as compared on day 21 and 35. Significant levels of IL-10 were detected on day 21 and 35 in Gp63 DNA vaccinated groups but not in both the polytope vaccinated groups. After challenge infection, rise in IL-10 cytokine was significantly lower in protein boost group of Gp63 DNA vaccinated animals. In case of Polytope DNA vaccines, though IL-10 increased after challenge infection but these levels were significantly low as compared to control group. There was no effect of protein boost on IL-10 levels in Poly/Hsp vaccine group (Fig. 4C).

In control (PBS) group, IL-4 levels on day 21 and 35 ranged from 32 to 50 pg/ml but on challenge infection, IL-4 levels were significantly enhanced. IL-4 cytokine levels were significantly low after challenge infection in all vaccine groups when compared to basal levels (controls) (p = 0.0001 to 0.007) minimum being in Poly/Hsp/PB and Poly/PB vaccinated groups. There was increase in these levels in unchallenged group on day63 for all the polytope vaccine groups and protein boosting did not significantly affect these levels (Fig. 4D).

### Parasite Load determination

**Figure 5:**
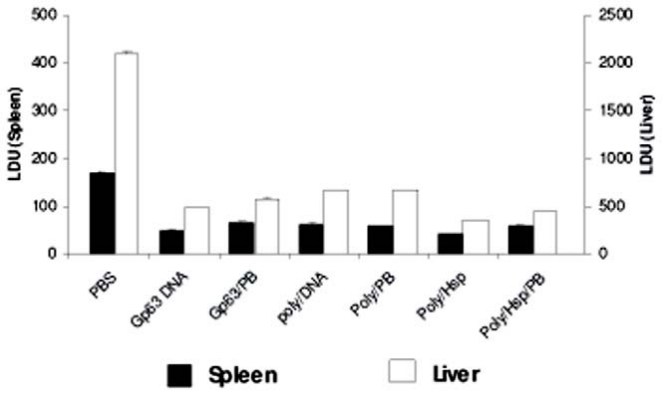
Parasite load in liver and spleen. All the vaccinated and control groups (as described in Fig. 2) were challenged intravenously (i.v.) with 2×10^7^ *Leishmania donovani* promastigotes on day35. Four weeks after the challenge, mice were sacrificed and Leishman Donovan units (LDU) were calculated from liver and spleen impression smears. The mean LDU ± SE is shown (n = 6) mice per group.

Efficacy of all the vaccine formulations was checked by determining the parasite load in both spleen and liver (Fig. 5). There was significant decrease in parasite load in both spleen and liver (p = 0.001 to 0.0001) after challenge infection in all the vaccinated groups for both single antigen and Polytope DNA vaccines when compared to the unvaccinated groups. But when all the vaccinated groups were compared, the parasite load was significantly lower in liver for Poly/Hsp vaccine group as compared to all other vaccinated groups. The parasite load in spleen was similar in all the vaccinated groups, minimum being in Poly/Hsp group which was significantly lower than that in Poly DNA (p<0.05). Protein boosting in any vaccination group did not significantly affect the efficacy of vaccines in terms of parasite load in both liver and spleen.

## Discussion

We demonstrate in our present study that epitope-based DNA vaccination represents a new vaccine strategy for *Leishmania* infection because of its excellent ability to induce T cell responses. DNA vaccine coding multiple continuous CTL or Th epitopes can induce specific CTL and Th responses to individual epitopes from different antigens[41], [42]. In our study, polytope DNA vaccine containing two T cell epitopes from Gp63 gene of *Leishmania donovani* and one universal Th epitope induced strong Th1 responses and much stronger cytotoxic responses than the single-antigen Gp63 DNA vaccination. Walker et al[43] showed that genetic immunization with glycoprotein63 cDNA from *L.major* resulted in Th1 immune response and protection in murine model of leishmaniasis. As reported by Button et al[44], *L. donovani* contains more chromosomal mini-exon gene sequences than *L. major* and this appears to be associated with increased virulence. Also VL (Kala azar) caused by *L. donovani* is a public health problem in several states of India. Therefore, we chose Gp63 of *Leishmania donovani* for the vaccine formulation. Curry et al[45] showed that PT7 epitope of GP63 of *L. major* stimulated IFN-γ production in majority of vervet monkeys recovered from *L. donovani* infection and IL-2/IL-4 production in all these animals. The present study shows that the two immunogenic epitopes in the coding region of Gp63 gene of *Leishmania donovani*, PT1 & PT7 [27] used for the preparation of polytope DNA vaccines induced Th1 type of immune response and strong cytotoxic responses. We believe that the stronger cytotoxic responses along with the higher IFN- γ levels generated by polytope/hsp DNA vaccine in our study may be attributed to CD8+ T cells of spleens isolated from vaccinated mice. Our data shows that these splenocytes were able to lyse *Leishmania* infected J774A.1 macrophages *invitro*. The perforin dependent pathway of cytotoxicity could have mediated this cytolytic activity of splenocytes particularly of CD8+ T cells which have been shown to be functioning in visceral leishmaniasis by Tsagozis et al[46]. Therefore, polytope vaccine approach provides an ideal strategy to improve the DNA vaccine's prophylactic efficacy. In our study, hsp70 molecule was used as genetic adjuvant to improve T cell responses since it has both cytokine and chaperone functions[47]. Li et al [3] showed that hsp70 was an intrinsic adjuvant molecule for polytope HBV DNA vaccine. Basu et al [48] showed in H-2b and H-2d mice models that hsp utilized the CD91R to be internalized by antigen-presenting cells (APCs), and the complexes of peptides with hsp90, calreticulin and hsp70 were also taken up by macrophages and dendritic cells and represented by MHC class I molecules Therefore, we assume that the hsp70-fused polytope antigen was internalized by receptor-mediated endocytosis and APCs presented the hsp-associated peptides, via their cell surface MHC class I molecules, to CD8+T cells; thus, antigen presenting function was improved. Interestingly, in present study the polytope DNA vaccine fused with hsp70 gene of *Leishmania donovani* provided an effective preventive strategy for visceral leishmaniasis as it enhanced the cytolytic activity of splenocytes isolated from vaccinated BALB/c mice and induced strong Th1 responses.

IL-10 is a major cytokine involved in progression of *Leishmania* infection to visceral disease[49]. IL-10 has been shown to block Th1 activation and consequently a cytotoxic response by down regulating IFN-γ levels and also because IL-10 inhibits macrophage activation, it decreases the ability of these cells to kill *Leishmania* [50]. Studies in humans on tissue cytokine mRNA expression have revealed that IL-10 is also involved in down regulating CD4+ T cell responses and disease pathology of *L. donovani* infections[51]. Because IL-10 usually exhibits human macrophage deactivating properties, high levels of IL-10 may represent a necessary counterbalance to an extremely polarized immune response thereby limiting the tissue damage[52]. In our study, though there was an increase in IL-10 levels after challenge in Poly and Poly/Hsp vaccine groups, the ratio of IFN-γ**:** IL-10 was still higher indicating the dominance of Th1 immune response.

As hypothesized, the polytope vaccine using hsp70 as an intrinsic adjuvant molecule resulted in significant reduction in parasite load after 4 weeks of challenge infection. To summarize, in the present study, both single antigen Gp63 DNA vaccine and polytope DNA vaccines with and without hsp molecule were found to be highly immunogenic as seen by splenocyte proliferation, cytotoxicity & cytokine production in vaccinated and control balb/c mice. Recombinant Gp63 protein boost enhanced the immunogenicity of single antigen Gp63 DNA vaccine as there was significant increase in Stimulation Index, Cytotoxicity and Th1 cytokine levels. There was no affect of protein boost on Polytope DNA vaccine. Both the vaccines showed significant efficacy against the challenge parasitic infection as seen by reduction in parasite load in balb/c mice. However, protein boost in any vaccine did not significantly enhanced the efficacy. When all the vaccines were compared, Polytope DNA vaccine with hsp70 showed highest Immunogenicity and efficacy.

To the best of our knowledge, this is the first study on polytope DNA vaccine for visceral leishmaniasis. Furthermore, we have successfully used the hsp70 gene from the same parasite species (*L. donovani*) in a polytope DNA vaccine while most previous studies on polytope DNA vaccines have employed hsp gene of mycobacterium or cancer cells[33], [53].

## Supporting Information

Data S1 Sequencing Result
(0.02 MB PDF)
