# Supplementary material for: Immunogenicity and Efficacy of Single Antigen Gp63, Polytope and PolytopeHSP70 DNA Vaccines against Visceral Leishmaniasis in Experimental Mouse Model
Source: PLoS One. 2009 Dec 2;4(12):e7880. doi: 10.1371/journal.pone.0007880 (PMC2780826; doi:10.1371/journal.pone.0007880)
Supplement: Data S1 — Sequencing Result (0.02 MB PDF) [file pone.0007880.s001.pdf]

ctagctgcgtTtacgGGCCCTCTAGACTCGAGCGGCCGCCACTGTGCTGGATATCTGCAG
AATTCCACCACaCTGGaCTAGT
GGTACCAAGCTTAAGTTTAAACCGCTGATCAGCCTCGACTGTGCCTT CTAGTTGCCAGCCATCTGTTGTTTG
CCCCTCCCCCGTGCCTTCCTTGACCCTGGAAGGTGCCACTCCCACTGTCCTTTCCTAATAAAATGAGGAAAT
TGCATCGCATTGTCTGAGTAGGTGTCATTCTATTCTGGGG
GGATCCGCGGCCATG AGACAGACACACTCCTGTTATGGGTACTGCTGCTCTGGGTTCCAGGTTCCACTGGT
GACATTGTGCTGACACAGTCTCCTGCTTCCTTAGCTGTATCTCTGGG GCAGAGGGCCACCATCTCATACAG
GGCCAGCAAAAGTGTCAGTACATCTGGCTATAGTTATATGCACTGGAACCAACAGAAACCAGGACAGCCAC
CCAGACTCCTCATCTATCTTGTATCCAACCTAGAATCTGGGGTCCCTGCCAGGTTCAGTGGCAGTGGGTCTG
GGACAGACTTCACCCTCAACATCCATCCTGTGGAGGAGGAGGATGCTGCAACCTATTACTGTCAGCACATT
AGGGAGCTTACACGTTCG GAGGGGGGACCAAGCTGGAAA GCCAAGTTCGTGGCCGCGTGGACCCTGAAGG
CCGCGGAGAAAGTGCGCGCCGCGAACTGGGGCGCGCTGCGCATCTCCGTCTCCAAA GCGGCGCGCTGCAT
CGATGGCGCCTTCAGGCCGAAGACGACCGAAACCAAA GGTGGGGTGGGGCAGGACag

Sequencing Result - Sequence of POLYTOPE DNA vaccine (561bp)
